# Methane Medicine: A Rising Star Gas with Powerful Anti-Inflammation, Antioxidant, and Antiapoptosis Properties

**DOI:** 10.1155/2018/1912746

**Published:** 2018-03-18

**Authors:** Yifan Jia, Zeyu Li, Chang Liu, Jingyao Zhang

**Affiliations:** ^1^Department of Hepatobiliary Surgery, The First Affiliated Hospital of Xi'an Jiaotong University, Xi'an, Shaanxi 710061, China; ^2^Department of SICU, The First Affiliated Hospital of Xi'an Jiaotong University, Xi'an, Shaanxi 710061, China

## Abstract

Methane, the simplest organic compound, was deemed to have little physiological action for decades. However, recently, many basic studies have discovered that methane has several important biological effects that can protect cells and organs from inflammation, oxidant, and apoptosis. Heretofore, there are two delivery methods that have been applied to researches and have been proved to be feasible, including the inhalation of methane gas and injection with the methane-rich saline. This review studies on the clinical development of methane and discusses about the mechanism behind these protective effects. As a new field in gas medicine, this study also comes up with some problems and prospects on methane and further studies.

## 1. Introduction

Methane, the simplest alkane, is the most plentiful organics on earth and has been studied for hundreds of years since its discovery in 1778. Being the main component of natural gas, methane is used as gas fuel. In past decades, it has been proven to be related to global warming since it contributes 20% of the greenhouse gases in the atmosphere and the concentration has raised rapidly [1]. In the clinic area, it was deemed antecedently that human bodies could not use methane. The endogenous methane is mainly excreted as flatus and it can also enter into the blood circulation and be exhaled by the respiratory system [2]. However, scientists recently reveal the biological effect of methane, especially the properties of anti-inflammatory, antioxidant, antiapoptosis and other clinic effects of methane, remains to be discovered.

There is about 200 ml gas in human enteric canal which is produced from various processes including air-swallowing, diffusion from blood, and biochemical reactions caused by bacteria in the enteric canal [3]. The proportion of healthy adult who can be detected with methane is more than 30%–50% worldwide [2, 4]. Anaerobic flora converts undigested carbohydrates into different organic compound including methane gas during fermentation [5]. To be more precise, methane is produced by a unique metabolic process, in which carbon dioxide is converted into methane with the hydrogen from anaerobic bacterial fermentation. *Methanobrevibacter smithii* and *Methanospaera stadmagnae* are the main methanogen in the intestinal tract, but in the oral cavity, *Methannobrevibacter oralis* is the chief methanogen and can lead to dental disease [6, 7].

## 2. Delivery of Methane

### 2.1. Inhalation

It is generally acknowledged that methane is a simple nontoxic asphyxiant, which means it is inherently nontoxic. Methane can be delivered via inhalation through many methods, including ventilator and facemask. As a flammable and explosive gas, the safe concentration of methane in pure oxygen is 4.9%. Nevertheless, methane should be used and stored with reliable tools and safety must always be the first concern. According to the study of Boros et al., the gas mixture of oxygen and methane (21% O_2_ + 2.5% CH_4_) is safe for rodents [8].

### 2.2. Injection of Methane-Rich Saline

Although inhalation is efficient and convenient, methane will bring the safety concern of explosion. Injection may make the delivery more safe and precise. A flexible way to produce a supersaturated methane-rich saline is set up through dissolving methane into sterilizing saline for 6 hours under the pressure of 0.4 MPa [9]. As methane gas is similar to hydrogen gas in chemical aspect, some researchers [10] used the same method to measure the concentration of methane as Ohsawa et al. described in hydrogen [11].

## 3. The Biological Effects of Methane

### 3.1. Liver

#### 3.1.1. Acute Liver Failure (ALF)

Acute liver failure (ALF) is the clinical manifestation of sudden and severe hepatic injury [12]. In the United States, over 6% of liver-related mortality was caused by ALF in 2005 [13]. Necrosis and apoptosis of hepatocytes induced by infection, chemical, or biological toxins are the dominant pathological causes of acute liver failure [13–15]. In a carbon tetrachloride- (CCl_4_-) induced acute liver injury mice model, methane showed a potential to be a therapeutic agent for ALF. Yao et al. showed that methane-rich saline could upregulate the expression of IL-10 by activating the PI3K/AKT/GSK-3*β* pathway, which would suppress the NF-*κ*B and MAPK pathways and raise anti-inflammatory properties [16].

#### 3.1.2. Autoimmune Hepatitis (AIH)

Autoimmune hepatitis (AIH) is a generally progressive chronic inflammation disease of the liver that occurs when the self-tolerance is broken down and hepatic cells are attacked by immune system accidentally [17]. The etiological and pathological mechanism of autoimmune hepatitis still remains unclear despite the genetic factor and environmental triggers, including infection, familial inheritance, and gender, are involved in the progress of the AIH [18]. Methane-rich saline showed kind of protection to concanavalin A-induced autoimmune hepatitis in the study of He et al. [19]. According to the study, the elevated serum aminotransferase levels in concanavalin A-induced autoimmune hepatitis mice model were reduced obviously after methane treatment. Furthermore, methane treatment reduced the phosphorylated I*κ*B, NF-*κ*B, and P38 MAPK in the liver, which consequently decreased the secretion of proinflammatory cytokines and increased the level of antioxidants.

#### 3.1.3. Hepatic Ischemia/Reperfusion (I/R) Injury

Hepatic ischemia/reperfusion (I/R) injury is induced by initial deficiency of blood supply to the liver and succeeding recovery of perfusion and oxygenation [20]. Surgery, transplantation, and circulation shock can all lead to liver I/R injury [21–23]. Ye et al. suggested that methane protects the liver against I/R injury through antiapoptotic, antioxidative, and anti-inflammatory actions by measuring inflammation makers, oxidant stress, and tissue injury [24].

### 3.2. Lung

#### 3.2.1. Acute Lung Injury (ALI)

Acute lung injury (ALI) is a destructive complication of several diseases such as acute circulatory failure, burn, and infection and is regarded as the main cause of acute respiratory failure [25]. ALI is clinically characterized by progressive hypoxemia and respiratory distress syndrome. The hallmark of ALI is injury to pulmonary capillary endothelial cells and alveolar epithelial cells and the activation of the innate immune, leading to diffuse edema in pulmonary interstitial and alveolar [26]. Sun et al. showed that methane-rich saline protected the lipopolysaccharide- (LPS-) challenged ALI via antioxidative, anti-inflammatory, and antiapoptotic effects, which had potential to be a new therapy for the treatment of ALI [27]. According to their results, it showed that the survival period was prolonged significantly after methane-rich saline treatment. The lung wet-to-dry (W/D) ratio and the number of inflammatory factors were reduced, and the levels of caspase-3 and apoptotic index were decreased either. In addition, methane-rich saline raised the antioxidants such as superoxide dismutase (SOD) and decreased the level of malondialdehyde (MDA) significantly, which proved the antioxidant property of methane.

### 3.3. Central Nervous System

#### 3.3.1. Spinal Cord Ischemia-Reperfusion (IR) Injury

Spinal cord ischemia-reperfusion (IR) injury is a destructive complication of several diseases such as spinal surgical procedures, hypotension, thoracoabdominal aneurysms, and thoracic [28]. The succeeding central nervous system injuries, such as paralysis, are severer health problems that have been continuously troubling patients [29]. The antioxidant, anti-inflammatory, and antiapoptotic properties of methane can also protect patients from spinal cord ischemia-reperfusion injury. Methane-rich saline (MRS) significantly decreased the level of inflammatory cytokines and oxidative products via the increased expression of nuclear factor erythroid 2 p45-related factor 2 (Nrf2) and downstream pathways related with the expressions of heme oxygenase (HO-1), SOD, catalase, and glutathione (GSH) at the onset of reperfusion. As a result of all these actions, neuronal apoptosis death was reduced and neurological function was preserved [10].

#### 3.3.2. Acute Carbon Monoxide (CO) Poisoning-Induced Injury

CO poison is an important cause of the accidental death. Methane protects brain from acute CO poisoning-induced injury with the properties of antioxidant, anti-inflammatory, and antiapoptotic. A finding suggested that methane reduced the level of inflammatory cytokines such as tumor necrosis factor-*α* (TNF-*α*) and interleukin1-*β* (IL-1*β*) in the brain but had no effect on interleukin 6 (IL-6) expression. In addition, the oxidative products such as malondialdehyde (MDA), 3-nitrotyrosine (3-NT), and 8-hydroxydeoxyguanosine (8-OHdG) were reduced after methane treatment while the amount of SOD in the hippocampus and cortex was decreased, which improved neuronal injury [30].

#### 3.3.3. Spinal Cord Injury

Spinal cord injury (SCI) and the subsequent risk of paralysis have been considered as a severe problem in clinic [31]. The USA statistics showed that the incidence of SCI reached 54 to 3393 cases/1 million in 2012, and mortality increased significantly when compared with that in 1993 [32]. There are various pathological mechanisms involve in SCI such as oxidative stress, inflammation [33], and apoptosis [34]. According to Wang et al. [35], methane can significantly decrease infarct area by reducing the pathological factor including oxidative stress, inflammation, and cell apoptosis following SCI. Additionally, the microglial activation can be significantly suppressed and hindlimb neurological function was preserved.

### 3.4. Immune System

The inflammatory disease is characterized as a pathological process caused by immune disorder including the dysfunction or excessive activation of immune system. Among the inflammatory diseases, sepsis and autoimmune colitis are regarded as serious problems clinically. The study of Zhang et al. [36] showed that methane-rich saline had the protective effect to inhibit some inflammatory signals caused by LPS in macrophages and suppress immune response in mice by intensifying IL-10 expression through PI3K/AKT/GSK-3*β* pathway. In conclusion, methane-rich saline treatment can alleviate endotoxin shock, bacteria-induced sepsis, and dextran-sulfate-sodium-induced colitis in mice.

### 3.5. Eye

#### 3.5.1. Retinal Ischemia/Reperfusion Injury (IRI)

Retinal ischemia/reperfusion injury (IRI) plays an important role in glaucoma, retinal vascular occlusion, diabetic, and many other diseases that can cause damage to human vision [37–39]. It can ultimately lead to blindness through neuronal damaging [40]. And in the pathological process of retinal IRI, retinal ganglion cells are the most susceptible and are regarded as the dominating factor. The study of Liu et al. [41] has shown that methane treatment was a promising therapeutic way for retinal IRI. According to their study, the level of oxidative products was reduced and the antioxidant enzyme was increased in retinas after methane treatment. Meanwhile, methane treatment obviously attenuated apoptosis in the retina by affecting the expression of the apoptosis-related gene and the caspase activity was limited as well. Thus, methane shows a protective role for the retinal ganglion cell (RCG) loss and dysfunction of vision in terms of retinal ischemia/reperfusion injury.

#### 3.5.2. Diabetic Retinopathy (DR)

Diabetic retinopathy (DR) is the main microvascular complication of diabetes whose succeeding problems such as blindness still remains to be serious problems in developed countries [42]. The inflammation [43], oxidative stress [44], and apoptosis [45] are involved in the pathology of diabetic retinopathy. The expression of TNF-*α*, IL-1*β*, glial fibrillary acidic protein (GFAP), and vascular endothelial growth factor (VEGF) in the DR retina were ameliorated after methane treatment. Moreover, the methane treatment upregulated retinal levels of miR-192-5p which is related to apoptosis and tyrosine kinase signaling pathway and also upregulated miR-335 which is related to proliferation, oxidative stress, and leukocyte. In terms of regulating miRNA, methane showed the protective effect on DR [45].

### 3.6. Motor System

The definition of overexercise is excessively prolonged or intense exercise, and many factors are associated with overload training including supercompensation and lack of recovery. Overexercise can lead to severe systemic disorders, such as rhabdomyolysis, acute kidney function failure, and systemic inflammatory response [46]. A study of Xin et al. showed that the methane-rich saline can promote the motor ability of rats such as treadmill running time and ameliorated exercise-related damage in gastrocnemius. At the meantime, the level of lactate acid and urea nitrogen in blood was reduced after methane treatment and the level of creatine kinase in plasma was decreased. Thus, methane may have a protective effect on the motor system in rats [47].

### 3.7. Cardiovascular System

#### 3.7.1. Myocardial Infarction (MI)

Myocardial infarction (MI) caused by coronary artery occlusion is the most common cardiovascular disease and a main cause of death worldwide [48]. It was found that methane-rich saline treatment can significantly ameliorate the apoptosis of cardiocytes and inhibit the subsequent myocardial remodeling. Thus, methane treatment can improve the cardiac function during the MI. And it is also found that the protective properties of methane-rich saline may be via its antioxidative, anti-inflammatory, antiapoptotic, and antiremodeling activities [9].

### 3.8. Skin

#### 3.8.1. Ischemia/Reperfusion (I/R) Injury-Induced Flap Loss

Skin flap transfer is a basic plastic surgery method, which is used widely in trauma surgeries and plastic surgeries. Some problems remain to be solved by skin flap transfer, and the most serious problem among them is I/R injury-induced flap loss [49]. According to previous studies, methane-rich saline may serve as a novel promising therapeutic agent for improving skin flap survival through the effects that suppressed apoptosis after transplantation and attenuate I/R injury. It was shown that a better blood perfusion with less inflammatory infiltration and cell apoptosis was established in the flaps after the treatment of methane and thus the survival area was increased significantly. Moreover, the apoptosis-related expressions including p-ASK-1, p-JNK, Bax, and caspase-3 activity were reduced by the methane treatment [50].

### 3.9. Gastrointestinal System

#### 3.9.1. Irritable Bowel Syndrome (IBS)

Methane was considered to be inert in biological field. However, more and more evidences have shown that methane is involved in many intestinal diseases and also be regarded as a detection of intestinal diseases according to the clinical data [51]. Irritable bowel syndrome (IBS) is a group of symptoms—including abdominal pain and changes in the pattern of bowel movements without any evidence of underlying damage and it can occur over years [52]. In different region, morbidity of IBS varies from 7% to 21% [53]. The cause of IBS still remains unclear but abnormalities occur in the gut flora, which happens after the infection is considered as a pathway of IBS [54]. Mechanism research has shown that methane takes part in constriction and velocity of the tract small intestinal and ileum. Moreover, studies in different animals have shown that methane can augment intestinal contractile function and subsequently slow the intestinal transit. Additionally, study in the guinea pig ileum showed that the peristaltic velocity was decreased and contraction amplitude was increased significantly after methane treatment [55–57]. In this way, researchers believe that methanogen and their products methane take part in the process of IBS.

#### 3.9.2. Intestinal Ischemia/Reperfusion Injury

The study of Boros et al. [8] provides evidence that methane inhalation can decrease the ischemia/reperfusion injury in intestine by involving in leukocyte activation and having a protective effect on oxidative and nitrative stress. According to the study, methane reduced the tissue damage index and the intestinal pCO2 gap. Moreover, methane treatment reduced the myeloperoxidase (MPO) activity and the intestinal oxidant stress levels. And also in the vitro experiment, the protective properties of methane were proven. Generally, the study shows that methane has properties of anti-inflammatory and antioxidant and has a potential to be a medicine for intestinal I/R injury.

#### 3.9.3. Acute Pancreatitis

Acute pancreatitis (AP) is a sudden inflammation in the pancreas and pancreatic acinar cell necrosis following the activation of pancreatin. The mortality of AP can be high. According to the study of Xie et al. [58], methane showed a protective property in cerulein-induced acute pancreatitis model. And the researchers further found out that the level of inflammation, oxidant, and apoptosis appeared to be reduced.

## 4. Discussion

Scientists have already revealed a few biological properties of methane in inflammation, oxidative stress, and apoptosis. Through these properties, methane can influence several pathological processes including I/R injury and sepsis. What is the exact mechanism underlying the protective properties of methane? The answer is unclear. So far, different researchers have come up with different hypotheses. Boros et al. [8] proposed that methane might accumulate at the interfaces of cell membranes and change the physicochemical properties or the situ functionality of proteins embedded in the environment. Kai et al. [59] assumed the membrane pathways including G protein, membrane, or receptor-mediated signaling and acetylcholine-activated ion channel kinetics may be involved in the mechanism that methane has shown in the previous studies. Fink [60] came up with several speculations to explain the biological effects of methane. In his speculations, cellular receptor, special oxygenase, and the formation of small amounts of the reactive alcohol, methanol, and/or changes in the redox milieu of the cell might be involved in the mechanism of the biological effects of methane. Since the mechanism of the protective effects is not clear, we discussed the protective properties of methane with an analysis of 15 studies (Table 1).

### 4.1. Anti-Inflammation

Inflammation is characterized by an increasing production of proinflammatory cytokines, leukocyte recruitment, and systemic and local regulation of leukocyte reactions [62]. A suitable balance of the inflammation process will lead to a defense reaction to the harmful target whereas the imbalance will lead to damage to the organism [63].

According to previous studies, inflammatory-related production during the tissue injury can be suppressed by methane treatment. Methane treatment influences some important pathway in activation of proinflammatory cytokines in lymphocytes and then regulates the cytokines. Yao et al. [16] showed that methane-rich saline may activate the PI3K-AKT-GSK-3*β* pathway to induce IL-10 expression and produce anti-inflammatory effects via the NF-*κ*B and MAPK pathways. Additionally, Wang et al. [10] indicated that methane reduced the level of inflammation by increasing the expression of Nrf2 and its downstream pathways.

### 4.2. Antioxidant

Oxidative stress is defined by an imbalance between the generation of free radical agent, like reactive oxygen species (ROS), and biological defenses that detoxify the free radical intermediates. The control of ROS production and antioxidant defense balance is necessary for normal cell function since oxidative take part in many pathological processes including I/R injury [64], cancer [65], and even neurological diseases [66]. ROS can initiate cell apoptosis or necrosis and the possible mechanism including DNA dissociation and lipid oxidation. Methane protects organism from oxidant in two aspects. On the one hand, it can raise the level of antioxidant factor such as SOD. On the other hand, methane can decrease oxidant factors like MDA and 3-NT. Moreover, the antioxidant effect may be related with the regulation of Nrf2 expression and oxidant-related miRNA like miR-335.

### 4.3. Antiapoptotic

Apoptosis is a process of programmed cell death that occurs in multicellular organisms and is important for homeostasis in multicellular life forms. In physiological manner, apoptosis diminishes harmed or transformed cells and needs for controlling of cell numbers, tissue, and organ morphology. Apoptosis is a highly regulated and controlled process that confers advantages during an organism's lifecycle. Lots of pathways are involved in the process of apoptosis, including Bcl-2/Bax and caspase system, and any disorder regulation of apoptosis often leads to cancer and tissue disorders. Song et al. [50] found that the level of Bcl-2 can be raised after the methane treatment. Additionally, the decline of JNK and ASK-1 showed to have the property to raise the level of Bcl-2 in the I/R mice model. Overall, methane shows an antiapoptosis effect by decreasing expression levels of activated ASK-1, JNK, and Bax and the increasing expression of Bcl-2. Here, we summarized indicators which were used to identify the properties of methane (Table 2).

### 4.4. Methane, Hydrogen, and Other Gases

Since Robert Furchgott, Louis Ignarro, and Ferid Murad shared the noble prize in medicine for their discoveries concerning “nitric oxide as a signaling molecule in the cardiovascular system” in 1998, more and more attention has been paid to the biological function of endogenous gases and lots of studies have confirmed that there are still many gases of important role in physiology in addition to nitric oxide (NO).

Carbon monoxide (CO) and hydrogen sulfide (H2S), two small molecules produced by human cells, are considered to have the similar function with NO in relaxation of vascular smooth muscle. NO can bind with the iron atom in the heme-containing protein and then change the protein to catalyze the guanosine triphosphate into the cyclic guanosine monophosphate which is also called the “second messenger” [67, 68]. The activation effect of CO is related with large-conductance calcium-activated potassium. However, the mechanism behind the activation of H2S is not so clear and adenosine triphosphate-sensitive potassium channel in vascular smooth muscle cells may be involved in the process [69].

Although NO, CO, and H2S have been regarded as the “star molecule” and has been studied most commonly, its chemical property is quite different from methane. Methane is quite unreactive and needs a critical condition, such as high temperature or ultraviolet. On the other hand, methane is a simple nontoxic asphyxiant for its unreactive characteristic but NO, CO, and H2S have more active property in biological field; in other words, they can be toxic sometimes.

In many aspects, hydrogen (H2) functions are in similar ways with methane. As a mild molecule, hydrogen can hardly disturb the normal reaction in the cell and also have a biological advantage by anti-inflammatory, antioxidant, and antiapoptosis [11, 70–72]. However, as more advanced researches about hydrogen have been done in past decades, hydrogen was found with more biological effects than methane and the mechanism of these effects is clearer than methane. Here, we list the comparison of methane and hydrogen (Table 3) and hope that more studies can be taken about methane in the future. As shown in Table 3, the delivery methods of hydrogen varied more. And the protective properties of methane in renal system and metabolic disease need to be demonstrated in the future. What is more, the mechanism behind these effects also needs to be clarified in the future.

### 4.5. Prospects of Methane

The study of biological function of methane develops rapidly. Methane, the most abundant organic compound on earth, was ignored in the medical field for decades. However, it has become a hotspot in therapeutic gas field. Recently, researchers have discovered some protective effect of methane and focus on the therapeutic function in I/R injury and inflammation disorder. Although lots of work has been done recently, there are still many problems unsolved. Firstly, the mechanism of several protective effects is still unclear despite different scholars have made different hypotheses. More pathways need to be detected. Secondly, since methane is effective in terms of being against inflammation, oxidative stress, and apoptosis, will methane become a potential medicine in cancer and other more diseases? Thirdly, according to the characteristic that methane is able to penetrate the cell membrane, can methane act as a signal molecule such as NO? Additionally, we also need to do a lot of research to formulate a standard that can provide the optimum dose, timing, and delivery methods. What is more, the disadvantage and toxicity of methane should be studied carefully before application.

## Figures and Tables

**Table 1:** The studies in this review.

Organs or systems	Animal models	Reference	Mechanism
Liver	(1) Carbon tetrachloride-induced acute liver injury	Yao et al. [16]	MS may activate the PI3K-AKT-GSK-3*β* pathway to induce IL-10 expression and produce anti-inflammatory effects via the NF-*κ*B and MAPK pathways Inflammatory cytokine: IL-6\TNF-*α*\IL-1*β*\IFN-*γ*\ICAM-1\CXCL1 ↓ IL-10 ↑ Inflammatory signal pathway: NF-*κ*B\p65\ERK\JNK\MAPK\P38 ↓
	(2) Concanavalin A-induced autoimmune hepatitis	He et al. [19]	Oxidant: MDA\8-OHdG ↓ Antioxidant: SOD\CAT ↑ Inflammatory cytokine: TNF-*α*\IFN-*γ*\IL-6\IL-1*β* ↓ IL-10 ↑ Inflammatory signal pathway: I*κ*B\NF-*κ*B\P38\MAPK ↓
	(3) Hepatic ischemia/reperfusion injury	Ye et al. [24]	Oxidant: MDA\8-OHdG ↓ Antioxidant: SOD ↑ Inflammatory cytokine: TNF-*α*\IL-6 ↓ Apoptosis: caspase-3 ↓

Lung	(4) Lipopolysaccharide-induced acute lung injury	Sun et al. [27]	Oxidant: MDA ↓ Antioxidant: SOD ↑ Inflammatory cytokine: TNF-*α*\IL-6 ↓ Apoptosis: TUNEL staining cells ↓

Central nervous system	(5) Spinal cord ischemia-reperfusion injury	Wang et al. [35]	MS increases the expression of Nrf2 and downstream HO-1, SOD, catalase, and GSH, inhibiting the production of inflammatory cytokine, oxidative products, and glial activation. Oxidant: MDA\3-NT\GSSG\H2O2 ↓ Antioxidant: HO-1\SOD\catalase\GSH ↑ Inflammatory cytokine: TNF-*α*\IL-1*β*\ICAM-1\CXCL1\MPO ↓ Inflammatory signal pathway: NF-*κ*B\p65 ↓ Apoptosis: caspase-9\caspase-3\MMP9 ↓ Nrf2 ↑
	(6) Acute carbon monoxide poisoning injury	Shen et al. [30]	Oxidant: MDA\3-NT\8-OHdG ↓ Antioxidant: SOD ↑ Inflammatory cytokine: TNF-*α*\IL-1*β* ↓ Apoptosis: caspase-9\caspase-3\MMP9 ↓
	(7) Spinal cord injury	Wang et al. [10]	Oxidant: MDA ↓ Antioxidant: SOD ↑ Inflammatory cytokine: TNF-*α*\IL-6\IL-1*β* ↓ Apoptosis: TUNEL staining cells\caspase-3 ↓

Immune system	(8) Endotoxin shock Bacteria-induced sepsis dextran-sulfate-sodium-induced colitis in mice	Zhang et al. [36]	MS limits LPS-induced NF-*κ*B/MAPK signal in macrophages and suppress immune response in mice by enhancing PI3K/AKT/GSK-3*β*-mediated IL-10 expression Inflammatory cytokine: TNF-*α*\IL-6\IL-1*β* ↓ IL-10 ↑

Eye	(9) Retinal ischemia/reperfusion injury	Liu et al. [41]	Oxidant: MDA\4-HNE\8-OHdG ↓ Antioxidant: SOD\CAT\GPX ↑ Apoptosis-related genes: bcl2 ↑ Bax ↓ Apoptosis: caspase-9\caspase-3\MMP9 ↓
	(10) Diabetic retinopathy	Wu et al. [61]	Inflammatory cytokine: TNF-*α*\IL-1*β*\VEGF\GFAP ↓ Apoptosis-related miRNA: miR-192-5p ↓ Oxidant-related miRNA: miR-335 ↓

Motor system	(11) One-time exhaustive exercise	Xin et al. [47]	Injury-related biomarker: CK\UN ↓ Antioxidant: T-AOC ↑ Inflammatory cytokine: TNF-*α*\IL-1*β*\IL-6 ↓ IL-10 ↑

Cardiovascular system	(12) Myocardial ischemia injury	Chen et al. [9]	Oxidant: MDA\8-OHdG ↓ Antioxidant: SOD\GSH ↑ Inflammatory cytokine: TNF-*α*\IL-1*β*\MPO ↓ Apoptosis-related pathways: Bcl-2/Bax\ASK-1/JNK Apoptosis: caspase-9\caspase-3\Bax ↓

Skin	(13) Skin flap ischemia/reperfusion injury	Song et al. [50]	Apoptosis-related pathways: Bcl-2/Bax\ASK-1/JNK Apoptosis: caspase-9\caspase-3\Bax\JNK\ASK-1 ↓ Bcl-2 ↑

Gastrointestinal system	(14) Intestinal ischemia/reperfusion injury	Boros et al. [8]	Oxidant: MPO\SOX ↓ NOx ↑
	(15) Acute pancreatitis	Xie et al. [58]	Inflammatory cytokine: TNF-*α*\IL-6\IFN-*γ*\IL-10 ↓ Oxidant: MDA ↓ Antioxidant: SOD ↑

**Table 2:** The indicators that are used to identify the properties of methane.

Pathology	Makers	Trend
Antioxidase	SOD CAT GSH	Increase

Oxidative stress	MDA 4-HNE 8-OHdG MPO 3-NT DAO	Decline

Inflammation factor	IL-1*β* IL-6 ICAM-1 IL-12 TNF-*α* IFN-*γ* CCL2 CXCL1	Decline

Inflammation signal	MAPK Lyn-P JNK ERK NF-*κ*B	Decline

Apoptosis	TUNEL Caspase-3 Caspase-9 Caspase-12 Bcl-2	Decline
Nuclear factor	Nrf2	Activation

**Table 3:** The comparison between hydrogen and methane in biological field.

Delivery methods	Hydrogen	Methane
	Inhalation of hydrogen gas [11] Oral ingestion by drinking hydrogen water [71] Injection of hydrogen-rich saline [73] Direct incorporation of molecular hydrogen by diffusion: eye-drop and bath [74, 75]	Inhalation of methane gas [8] Injection of methane-rich saline [9]

The biological effects [76]	(1) Protective effects against reperfusion injury (2) Protective effects against neurodegeneration (3) Preventive effects against metabolic syndrome (4) Suppressive effects on inflammation (5) Mitigation of side effects in treatments of cancers (6) Effects on dermatomyositis and mitochondrial diseases (7) Mitigating effects of hemodialysis (8) Effects on acute erythematous skin diseases (9) Effects on exercise	(1) Protective effects against reperfusion injury [8, 9, 24, 27, 35, 41, 50] (2) Suppressive effects on inflammation [16, 19, 27, 36, 58] (3) Effects on diabetic complication [41] (4) Effects on exercise [47] (5) Protective effects on carbon monoxide toxicity [30]

Mechanism [76]	(1) Direct reduction of hydroxyl radicals with molecular hydrogen (2) Direct reduction of peroxynitrite with molecular hydrogen to regulate (3) Indirect reduction of oxidative stress by regulating gene expression (4) Regulation of gene expression of proinflammatory cytokines and hormones (5) Regulation of other genes (6) Modulation of signaling	(1) Direct reduction of inflammation-related pathways (2) Direct reduction of oxidant-related pathways (3) Direct reduction of apoptosis-related pathways (4) Regulation of gene expression of oxidant-related genes
